# Exploring the lunar water cycle

**DOI:** 10.1073/pnas.2321065121

**Published:** 2024-12-16

**Authors:** Philipp Reiss

**Affiliations:** ^a^Technical University of Munich, School of Engineering and Design, Department of Aerospace and Geodesy, Ottobrunn 85521, Germany

**Keywords:** Moon, volatiles, water, exploration, solar system

## Abstract

The presence of water on the Moon has been indicated by various remote-sensing observations and analyses of returned samples. Several missions are planned to conduct new in situ research on the lunar surface to directly observe and characterize lunar water. A comprehensive characterization of the present forms of water, their abundance, spatial distribution, temporal variation, and possible origin is required to understand the lunar water cycle and the relevance of individual source and sink mechanisms and transformations between the involved volatile species. These processes extend over vastly different scales, and the governing parameters are often insufficiently constrained by experimental and observational data. Here, I present a brief overview of the current state of knowledge on the lunar water cycle, its relevance for lunar science and exploration, and some of the main challenges of modeling and future in situ analyses aiming to substantially advance the understanding of lunar water occurrences.

Water plays an important role in the history and evolution of the solar system, and its presence on the Moon was discovered only a couple of decades ago. As an airless body, the Moon preserves many interactions with the space environment over geological timescales, including solar wind and impact bombardment, thus a continuous delivery, formation, and loss of volatile components. This leads to the evolution of a complex inventory of volatiles directly accessible at or near the surface. Among these, water is of particular interest, mainly due to its utilization potential as a consumable for life support and propellant for space exploration missions, and because its form, abundance, and variability are closely related to the evolution of the Moon and its interaction with the environment. The relevant forms of lunar water discussed in this context include molecular water (H_2_O), hydroxyl (OH), and forms of water ice, while occurrences range from adsorbed, mineral-bound, or trapped species to granular mixtures with regolith. Indigenous water is found in minerals and glasses and is directly linked with the formation of the Moon. Until the present day, exogenous water has been introduced through interaction with the solar wind and by the impact of dust particles, asteroids, and comets. The lunar water cycle described here focuses on the highly volatile species mainly stemming from exogenous sources.

Evidence for the existence of lunar water has been delivered through remote observations by various missions over the past decades, summarized in Table 1. These include Clementine (1), Lunar Prospector (2), Cassini (3), Deep Impact (4), Chandrayaan-1 (5, 6), Chandrayaan-2 (7), and the Lunar Crater Observation and Sensing Satellite (LCROSS) with the Lunar Reconnaissance Orbiter (LRO) (8, 9). Other remote data were collected by the airborne Stratospheric Observatory for Infrared Astronomy SOFIA (10, 11) and the Infrared Telescope Facility IRTF in Hawaii (12). Studies of returned lunar samples provided direct evidence for the existence of indigenous or solar wind-induced lunar water (13–15). Several hypotheses have evolved from these measurements to explain the form, abundance, spatial distribution, temporal variability, and possible origin of water in the lunar environment (16–20). To date, many of the governing processes and parameters are still associated with large uncertainties, necessitating primarily new in situ measurements in conjunction with improved models, analog experiments, and sample analyses to advance our understanding of the lunar water cycle.

**Table 1.** Overview of evidence for water at the lunar surface based on remote observations

Mission	Technique	Measurement	Derived H_2_O concentration (by mass)
Clementine	Radar	Bistatic radar, magnitude, and polarization of the radar echo versus bistatic angle	inconclusive
Lunar Prospector	Neutron spectroscopy	Epithermal and fast neutrons, resulting from collisions of cosmic ray neutrons with H	1.5 ± 0.8% (21)
Cassini	Visible/Near-infrared spectroscopy	Reflectance spectroscopy, absorption feature at ~3 μm for OH or H_2_O	10 to 1,000 ppm (3)
Deep Impact	Visible/Near-infrared spectroscopy	Reflectance spectroscopy, absorption feature at ~3 μm for OH or H_2_O	<0.5% (4)
Chandrayaan-1	Visible/Near-infrared spectroscopy, Mass spectrometry	Reflectance spectroscopy, absorption feature at ~3 μm for OH or H_2_O, overtones, and combination mode vibrations for H_2_O near 1.3/1.5/2.0 μm	<770 ppm (5); 500 to 750 ppm (22); <30% (23)
Chandrayaan-2	Near-infrared spectroscopy	Reflectance spectroscopy, absorption feature at ~3 μm for OH or H_2_O	<800 ppm (7)
Lunar Reconnaissance Orbiter	Ultraviolet/Visible/Near-infrared spectroscopy, proton flux, ultraviolet spectroscopy, neutron spectroscopy, radar	Reflectance spectroscopy, absorption feature at ~1.87 μm for H_2_O vapor, energetic proton albedo, Lyman-alpha line, epithermal neutrons, radar circular polarization ratio	5.6 ± 2.9% (8); 1 to 2% (24); 0.1 to 2.0% (9); 0.9 to 4.9% (25); 5 to 10% (26)
Stratospheric Observatory for Infrared astronomy	Near-infrared spectroscopy	Reflectance spectroscopy, absorption feature at ~6 μm for H_2_O	100 to 400 ppm (10); <170 ppm (11)
Infrared Telescope Facility	Near-infrared spectroscopy	Reflectance spectroscopy, absorption feature at ~3 μm for OH or H_2_O	<700 ppm (12)

The spatial scales on which remote measurements resolve the detected forms of lunar water are very different, and the sensing depths range from tens of micrometers for infrared spectroscopy over decimeters for neutron spectroscopy to tens of meters for radar measurements. Furthermore, these sensing techniques are sensitive to different species. Neutron spectroscopy is sensitive to hydrogen-bearing species, but it cannot discriminate between different forms of hydration, such as H, OH, or H_2_O. Epithermal neutron flux provides a good measure of the hydrogen content in the lunar soil, and by comparison with the fast neutron flux, the burial depth of hydrated material can be derived within certain limits (27). The spatial resolutions achieved by past measurements of the neutron flux are typically on the order of tens of kilometers (28). Infrared spectroscopy mainly yields the spectral absorption feature in the 3 μm region as an indicator for water, but because both OH and H_2_O exhibit fundamental O–H vibration in this range, they are difficult to discriminate. Additionally, thermal emissions can interfere with these absorption features at higher wavelengths. Thermal correction is typically applied to compensate for this, and a broader spectral range, such as provided by the instruments on Deep Impact, Chandrayaan-2, and at the IRTF, helps to reduce uncertainties (7, 12). The highest spatial resolution datasets regarding lunar water were obtained by the Imaging Infrared Spectrometer on Chandrayaan-2 with ~80 m across a spectral range of 0.8 to 5.0 μm (7) and the Moon Mineralogy Mapper M^3^ on Chandrayaan-1 with 140 m at 0.43 to 3.00 μm (5). In comparison, the SpeX spectrograph of the IRTF provided a resolution of 1 to 2 km at 1.67 to 4.20 μm (12). Nighttime surface temperatures derived from the LRO Diviner Lunar Radiometer Experiment measurements currently achieve an effective resolution of ∼330 m longitudinally and ∼700 m latitudinally at the equator (29). The most recent datasets are binned to a resolution of 128 pixel-per-degree, which corresponds to approximately 240 m and to 0.25 lunar hour time steps (30). Variations on a smaller spatial and temporal scale, especially in the polar regions, can so far only be approximated by thermal models.

Water mass fractions derived from reflectance spectroscopy typically lie in the range of several hundred parts per million, as shown in Table 1 (3–5, 7, 10–12, 22, 31). By investigating overtones and combination mode vibrations for H_2_O near 1.3, 1.5, and 2.0 μm, Li et al. (23) derived much higher local mass fractions of up to 30%. Concentrations detected in polar regions are distinctly higher than toward the equator, mainly owing to the different thermal conditions at the surface (20). The detected forms of water may also vary strongly between these regions, with adsorbed water predominant in the polar regions and implanted or trapped water closer to the equator (32). Absorption features in reflectance spectroscopy are interpreted by comparing them with modeled or empirical spectra. The absorption features indicative of water strongly depend on its physical state, the type of mixing with regolith, grain sizes, temperature, albedo variation, multiple scattering, and viewing geometry (3, 22, 23), and thus deriving definite water concentrations from such orbital measurements is challenging.

The only estimate of lunar H_2_O concentration in a permanently shadowed region close to ground truth is the data acquired by the LCROSS impact in the crater Cabeus, where a mass fraction of 5.6 ± 2.9% was derived from the observation of impact ejecta (8). The uncertainty of this measurement stems from the near-infrared spectroscopy of the impact plume and the estimation of the ejected debris mass of several tons using brightness measurements of the ultraviolet–visible spectrometer. The original form, spatial distribution, and depth distribution of the excavated water cannot be derived from the data. Some of the excavated water might have been trapped in the impact crater or ejected around the impact site and thus would not have been detectable in the plume. Nonetheless, the order of magnitude of the derived water mass fractions is supported by other instruments onboard the LRO, which analyzed the ultraviolet albedo of polar cold traps [1 to 2% (24), 0.1 to 2.0% (9), 0.9 to 4.9% (25)] and performed radar measurements [5 to 10% (26)]. Earlier neutron flux measurements by Lunar Prospector yielded similar mass fractions of 1.5 ± 0.8% near the south pole (21).

The analysis of indigenous water in returned samples allows more complex measurements than possible by remote sensing. Data derived from a variety of samples retrieved by the Apollo, Luna, and Chang’e missions, as well as lunar meteorites, provided accurate information on the water concentrations with several hundred to thousand parts per million by mass, depending on the type of sample and associated source of water (14, 15, 33). The measured deuterium to hydrogen (D/H) isotope ratios hint at a volcanic, cometary, or solar wind origin, whereby mixtures of these origins also need to be considered, such as the transfer of solar wind implanted hydrogen to agglutinate glass via impact melting. However, several processes lead to an alteration of the original D/H ratio, including the outgassing of magmatic water from the melt, the reduction of water into hydrogen, or the production of deuterium via cosmic-ray spallation reactions (14, 34, 35).

The lunar water cycle constitutes various source, sink, and loss or conversion mechanisms, as described in the following (20, 32). Elements delivered by the solar wind and implanted into the upper layers of the lunar regolith are a weak yet continuous source of volatiles, as solar wind protons react with dangling oxygen bonds to create OH and H_2_O (36, 37). In the early history of the Moon, the extensive impact bombardment with the delivery of hydrated minerals and ice was by far the strongest source of water, with up to four orders of magnitude higher contribution as compared to the production of water induced by the solar wind (38). Internal outgassing of the early Moon also contributed to the accumulation of water, although there is some uncertainty as to what extent the volcanic activity could have led to the formation of polar water deposits (39, 40). Another volcanic contribution was the explosive eruption of basaltic magma, which formed and dispersed pyroclasts at the surface, containing trapped water and other volatiles (41). Sink mechanisms for lunar water involve cold trapping through molecular adsorption, melt trapping through gas inclusion, and the burial of ice deposits by regolith. Loss or conversion mechanisms include impact vaporization and thermal release, photolysis including photon-stimulated desorption and photodissociation, temperature-driven (potentially recombinative) desorption, atom sputtering, and thermal escape of free molecules. Water molecules migrate across the lunar exosphere through ballistic hops and interaction with electromagnetic fields. Within the subsurface, molecular migration is driven by thermal desorption and adsorption, as well as diffusion processes.

The aforementioned mechanisms have similar implications for other species present in the lunar environment, such as the noble gases helium, neon, and argon (42), and on other airless celestial bodies, such as Mercury, the Martian moons, asteroids, comets, dwarf planets, as well as the (icy) moons of the outer solar system (43, 44). Examples are the carbon dioxide exosphere observed at the Galilean moon Callisto (45) and in the Uranian system (46, 47), and refractories like calcium and magnesium and alkali metals like sodium and potassium in the exosphere of Mercury (48, 49).

## Relevance for Science and Exploration

Understanding the lunar water cycle is relevant for very different areas of research, such as studying the interaction of the Moon with its space environment, assessing the alteration of the lunar environment through surface activity, developing instrumentation for future in situ measurements, and exploring and potentially utilizing lunar water as a resource.

### Interactions with the Space Environment.

By studying the accumulation of water and the retention dynamics on the lunar surface, the flux and composition of external sources can be qualitatively traced back in time. The mechanical mixing of regolith by impacts causes the release of volatile species, but also the burial of exposed surfaces in the vicinity, shielding these from further solar wind and galactic cosmic ray interaction and preserving their composition. Coverage of the regolith surface by volcanic lava flows is another mechanism to preserve exposed rocks and soil. By investigating implanted elements at different depths and relating these to the exposure, or soil maturity, the evolution of the past flux and composition of the solar wind or galactic cosmic rays can be derived, as well as the extent of soil mixing due to impacts (50, 51). The main difficulty lies in determining the possible alteration of volatile elements due to the interaction of different external sources, interactions with the grain surface, and the thermal effect of impacts and lava flows. Estimating the rate of coverage by impact events is an additional challenge, as the depth of an ejecta blanket changes with distance and can be discontinuous, as in the case of crater rays. Crider et al. (52) showed that hydrogen concentrations in the subsurface reach a saturation limit over time, where the delivery by the solar wind is balanced with the loss via continuous small impacts. At the same time, the layers with enriched hydrogen concentration migrate deeper over time. Discrete events that deliver larger amounts of water, such as the impact of a larger comet, might not only lead to a concentrated deposition of water in the vicinity of the impact but can also cause a global collisional atmosphere which remains stable over several Earth days (53). Such a drastic change in the environmental conditions strongly affects the dynamics of water migration and deposition. A better understanding of such events and surface interactions will allow for reconstructing the past evolution of lunar water occurrences.

### Alterations Due to Surface Activity.

Future lunar surface activity will undoubtedly change the natural lunar environment and cause an alteration and loss of the pristine regolith and volatile properties. This will affect the ability to study related processes that have been active over geological timescales. Weakly bonded water molecules at the grain surface will likely be chemically altered or released, mainly through thermal interaction and reaction with other volatile species. For instance, the powered descent of a lander can release surface water, which is subsequently lost to space but, to some extent, migrates to cold traps over the course of several lunar days (54). Stronger bonds might be less affected, as shown by recent studies on solar wind derived water found in samples returned by the Chang’e 5 mission (15). Being mainly concentrated in the amorphous rims of grains, a small amount can diffuse into the crystal lattice and be preserved there. The sensitivity of all forms of pristine water to alteration needs to be investigated at various scales, and it is important to determine how far the disturbance of lunar surface activity reaches and to what extent the respective release, transport, and deposition processes are affected. Assessing these interactions is crucial to developing possible mitigation strategies, such as adapting operational planning to account for the protection of areas sensitive to the release of volatiles or imposing restrictions on the use of specific spacecraft materials and propellants. Understanding the lunar water cycle and the contributions of surface activities thus is important to inform the management of future scientific and commercial exploration and resolve potential conflicts (55).

### Future In Situ Measurements.

The development of instrumentation and the prediction and interpretation of measurement data are strongly influenced by the interaction with the environment. For instance, molecules released by local outgassing events, either from a spacecraft or a warm sunlit surface area, will be preferentially detected by an instrument pointing toward this source. A temporary collisional exosphere that develops due to surface activity (56) or through the thermal release of volatiles triggered by the passing of the terminator will cause transient changes in the exosphere column density. Surface topography, as well as the resulting thermal environment or the local distribution of existing volatile occurrences, affect the extent to which an instrument can detect or resolve new volatile release events. Instrument and spacecraft contamination also contribute to the local exosphere and need to be accounted for. Possible effects are the saturation of instrument detectors, such as reported by Stern (57) for the Apollo cold cathode gauge experiments. Lucey et al. (20) suggested optical detector saturation to explain why water desorption observed at ultraviolet and infrared wavelengths exhibits a different characteristic. The positioning and operation of scientific instruments for water prospecting are typically constrained by the landing site and its vicinity, and site selection is based on remote sensing datasets for surface features, temperature, illumination, and slope angles. Most of these datasets are based on reflectance spectroscopy and only refer to the top surface of the regolith. Augmenting the existing datasets with information on transient water migration, both above and below the surface, might reveal new potential landing sites and traverse routes and thus increase the opportunities for the scientific characterization of water occurrences.

### Exploration and Utilization of Resources.

Trends in global water migration predicted by simulations will support the identification of the resource potential, associated mining sites, and planning for in situ resource utilization operations (58). Planned lunar outposts will rely on this capability, and any process related to in situ resource utilization downstream the value chain depends on the properties of a water reserve. The recent orbital prospecting missions (Table 1) only provide an indication and first measurement of lunar water deposits. Further in situ exploration is needed to analyze and characterize these deposits to increase confidence in the exact locations, depths, concentrations, and forms of water. The result of this evaluation determines whether a resource constitutes a reserve, meaning that it can be economically and legally extracted (59, 60). Understanding the lunar water cycle is important to support this resource exploration and the assessment of its reserve potential. Extracting and utilizing water on the Moon is an essential prerequisite for long-term human and robotic exploration, as producing consumables and propellant in situ is more economical and flexible than a regular resupply from Earth.

## The Challenges of Modeling the Lunar Water Cycle

The above considerations highlight the need for simulation models in support of and as an extension of in situ data. Most of the current models on the lunar water cycle describe the source, sink, and loss or conversion mechanisms in their individual domains. The segregation into separate models is mainly due to the complexity and vastly different spatial scales of the individual processes, such as the transport across the exosphere, surface, and subsurface, as well as transformations between species and surface reactions. This limits the current capabilities to investigate the lunar water cycle and interpret future in situ observations. Besides the computational model complexity, many model parameters are presently not available or are associated with a high uncertainty. More in situ and laboratory-based measurements are therefore needed to enable model-based sensitivity analyses and identify the governing processes for the lunar water cycle and their key parameters.

Computationally, the biggest challenge for a substantially broader simulation is the high number of processes acting on vastly different scales (61). For example, topographical features are typically represented on local to global scales, and molecular diffusion usually requires grain-level resolutions. The problem is reduced for a body with a collision-less exosphere as the lack of convective fluxes reduces the number of phenomena to model. Nonetheless, the fundamental problem of scales persists, and the most straightforward approach is to assess the sensitivity of the models to certain parameters and, based on this, reduce the model complexity by simplifying less relevant parameter dependencies. This includes prioritizing and potentially combining the effect of loss processes such as ion sputtering, photon-stimulated desorption, electron-stimulated desorption, or micrometeoroid impact vaporization. Knudsen diffusion between the grains, occurring at low gas density when the pore space is smaller than the mean free path and molecules collide more frequently with the grains than with other molecules, and surface diffusion along a grain surface driven by molecular jumps between adjacent free adsorption sites, could be expressed in a combined diffusion coefficient. Other simplifications include an optimized computation approach, where the surface topography is studied a priori to derive probability distributions of roughness and slope on a coarser grid before running the coupled simulation, thus potentially saving a significant amount of simulation time.

For modeling water retention at different scales, one of the key aspects is surface roughness. Hayne et al. (62) estimated the effect of small-scale shadows in the range of 1 km to 1 cm and found that cold traps on a scale of 1 cm are the most abundant. By including shadowing effects on such scales, a significantly larger number of smaller regions with potential water stability, so-called “micro cold traps,” were found (62, 63). Prem et al. (64) showed that surface roughness on the order of 20° (root mean square) based on the results of Bandfield et al. (65), especially near the poles, causes an increase in the global cold-trapping of water. The accumulation of water in areas with more small-scale cold traps also increases the probability of the water being mobilized, thus likely enhancing the poleward migration of water molecules. The dynamic thermal environment above the surface is a key element in the overall migration of water, as it has a nonnegligible effect on the stability of ice at different depths. Temporary sequestration of water causes a complex migration pattern, especially at high latitudes (66), and smaller concentrations of adsorbate can remain stable in the shallow subsurface even if the temperature above temporarily exceeds the threshold for its release (67). Water migrates within the subsurface through the interaction of thermal desorption and diffusion along concentration gradients, a process called thermal pumping (68, 69), and strongly depends on the surface heat flux. Localized enhancements of water concentration detected by SOFIA further support the hypothesis that small-scale shadowing plays an important role in water retention at the surface (11). Neglecting these small scales when modeling water migration will lead to underestimating water retention, especially at high latitudes with shallow solar incidence angles. For models based on surface temperature maps, the topographic effects are indirectly included to some extent because the observed temperature is affected by surface scattering and shadowing. Smaller-scale roughness also has an effect on the ballistic trajectories of exospheric elements and locations with distinct slopes and surface temperatures might cause local variations in the column density as molecules are released into preferential directions.

An additional challenge is the sparsely available data in the literature, for example, on parameters such as diffusion or surface reaction rates. The resulting uncertainty unavoidably affects the quality of the simulation results, but the severity of this impact also depends on the fundamental sensitivity to the respective parameter. Examples of parameters with higher uncertainty are the regolith particle size and morphology, which translates into the tortuosity of a packed regolith bed and ultimately into the molecular diffusion coefficient, thus the overall mass transfer through regolith. Directly measuring the diffusion coefficient of water is challenging, especially at lower temperatures, where adsorption on the grain surface occurs. Empirical data only exist for higher pressures and temperatures, such as the work of Schieber et al. (70), Schieber et al. (71), and Farr et al. (72), who evaluated an advection–diffusion model for the transport of nitrogen, argon, and water vapor through lunar regolith across different flow regimes. Temperatures in these experiments ranged from room temperature to 350 K and gas pressures from 50 Pa to 25,000 Pa. Thus, the Knudsen regime relevant to most water migration processes in lunar conditions was only considered to a very limited extent, up to approximately 100 Pa in this study. Another larger uncertainty stems from the sorption kinetics and the associated binding energies. Poston et al. (73) found a significant variation of adsorption energies between mare-type regolith (peak at 0.5 eV) and highland-type regolith (peak around 0.7 eV and up to 1.5 eV), with highland-type regolith retaining up to 40 times more water. The exponential dependence via the Arrhenius law can cause variations of many orders of magnitude in the resulting desorption rate.

The retention and transformation of chemical species via reactions in the exosphere and at the surface has proven to be a crucial factor in determining the exospheric composition (74–77). It is only possible to reproduce the observed trends in hydrogen concentration by including conversions between H, H_2,_ OH, and H_2_O and taking into account the associated binding energies and loss rates. Larger uncertainties, however, remain regarding the temperature-dependent reaction rates of the regolith-bond species to exospheric H and H_2_O and the conversion rates of the latter via photolysis or photoexcitation. A further contributor to hydrogen conversions is methane, which is assumed to form via the reaction of solar wind implanted carbon with hydrogen (78–80). However, with a peak value of 450 cm^–3^ observed at a reference altitude of 12 km, its concentration is at least one to two orders of magnitudes lower than for hydrogen (81). A closer investigation of the transformation pathways also involves a better distinction between the actual concentrations of H_2_O and OH present at the surface. In addition to the molecular adsorption at the topmost grain surfaces, molecules can scatter and diffuse into deeper grain layers before being released back to the exosphere. As a result, the effective residence time of volatiles at the lunar surface can be much longer than when only considering adsorption at the topmost surface (82), and the resulting delay needs to be considered when reproducing observational data.

The uncertainty of microphysical soil properties is even higher for ice–regolith mixtures, and higher concentrations of ice can have a significant impact on the heat and mass transfer. The conditions under which ice forms and the resulting ice geometry determine the bulk density, porosity, tortuosity, and specific surface area and can lead to very different properties. Siegler et al. (83) found that large temperature gradients between grains at low gas pressure preferentially cause the growth of anisotropic ice tendrils between semidistant grains, as opposed to isotropic neck structures between neighboring grains. This leads to a linear increase in thermal conductivity with ice content and can also cause a chain-like agglomeration of particles. Reduced porosity and increased tortuosity inhibit mass transfer through the voids, and the effect of ice growth needs to be better understood to improve respective models. For the experimental investigation of ice–regolith mixtures, the main challenge lies in preparing analog samples while maintaining reproducibility and representativity. Applied methods for past experimental studies include mixing regolith with liquid water (84) or hydrated mineral salt (85), adding water vapor or dispersed water to regolith (86), freezing droplets of dust particles dissolved in water (87), exposing regolith to a humidified carrier gas (88–91), and injecting water vapor into an evacuated chamber with regolith (92, 93). The water mass fractions achieved in these samples range from less than 0.1% for the adsorption studies to 75% for the dust particles dissolved in water.

## Predicting the Lunar Ice Stability

Despite the complexity of the lunar water cycle and the aforementioned challenges in modeling, the presence of ice at a respective location is typically predicted using a highly simplified approach based on threshold values for its sublimation temperature. The most popular approach was introduced by Paige et al. (94), who arbitrarily chose a sublimation rate of 1 mm m^–2^ Ga^–1^ as a boundary to distinguish whether water ice is stable or not. Given the age of the Moon of roughly 4.5 Ga, the associated maximum loss of ice at this rate is negligible. The sublimation rate is calculated based on the saturation vapor pressure (69, 95), described by the Clapeyron equation, which matches well with empirical data (96, 97). Assuming a constant ice density of 1,000 kg m^–3^, thus a sublimation rate of 1 kg m^–2^ Ga^–1^, Paige et al. (94) defined a threshold temperature of 101.3 K, referred to as the “volatility temperature.” It should be noted that when using a more representative value of 933 kg m^–3^ for the density of hexagonal ice (98), this definition of the threshold temperature only decreases by 0.1 K. When assuming a more realistic increase in ice–regolith density over depth, based on the model of Carrier et al. (99) for pure regolith, the threshold temperature changes to 100.6 K. A significantly higher value of 110 K is commonly cited in the literature as a basis to assess ice stability at a certain location (9, 23, 32, 100, 101). However, the sublimation rate at 110 K for a constant density of 933 kg m^–3^ is 121 mm m^–2^ Ga^–1^, two orders of magnitude higher than the threshold value originally introduced by Paige et al. (94).

**Fig. 1.**
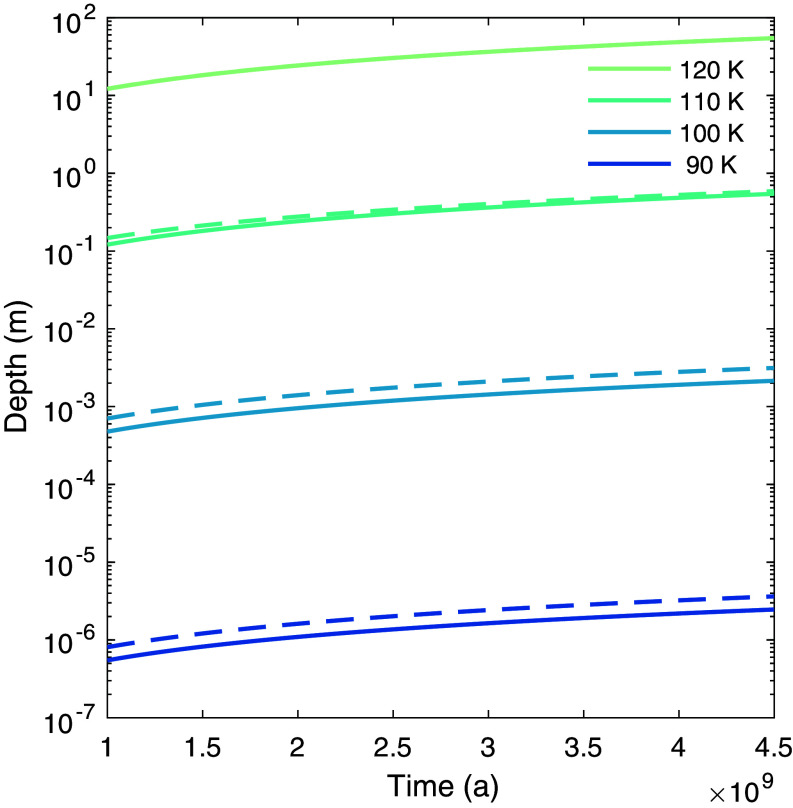
Sublimation depth over time of pure water ice for different constant temperatures. A constant density of 933 kg/m^3^ (solid lines) and a hyperbolically increasing density over depth (dashed lines) are depicted for comparison.

Fig. 1 shows the strong sensitivity of ice stability against temperature and displays that it is crucial to understand the implications of temporal and spatial temperature variations, as well as temperature measurement and prediction uncertainties. For cases where the temperature exceeds a certain threshold only during a fraction of the time, ice stability needs to be assessed depending on the changing thermal environment. This also means that uncertainties associated with temperature data from remote sensing and model predictions strongly affect the derived ice stability. In addition, when deriving the present-day concentration from past evolution, for instance by applying sublimation rates, the time of deposition and the initial amount of ice must be considered. Ice that formed later in the history of the Moon, e.g., through impact delivery a few million years ago, might still be present today, even if the surface temperature is above the volatility temperature. Thus, the quantity and distribution of ice occurrences are likely underestimated when applying a fixed temperature threshold as described above. Vice versa, a more comprehensive investigation of the water cycle might allow the definition of more flexible criteria to define ice stability and potentially reveal more abundant water occurrences than currently possible.

## The Challenges of In Situ Analysis

Addressing the current knowledge gaps and reducing model uncertainties requires more in situ analysis on the surface of the Moon to better constrain model parameters. Many space agencies are currently pursuing the goal of finding and characterizing lunar water in situ to provide the first ground truth for previous remote observations (Table 2). A significant challenge for these missions is the accessibility of locations with near-surface water occurrences, as these will predominantly be found in a very cold environment with rough terrain and limited illumination. Furthermore, the typical spacecraft limitations in mass, power, and volume also translate to the instrumentation onboard and lead to a trade-off between these resources and the analytical capabilities. For mobile rovers, the resource limitations are often even stricter than for landers, which leads to another potential trade-off between spatial coverage and resolution of the acquired data.

**Table 2.** Planned missions relevant to the in situ prospecting of lunar water

Mission	Platform	Instruments relevant to water prospecting
PRIME-1	Lander	Mass spectrometer (102)
LUPEX	Lander, rover	Thermo-gravimetric analyzer, mass spectrometers, cavity ring-down spectrometer, Raman spectrometer, infrared spectrometer, neutron spectrometer, ground-penetrating radar, permittivity, and thermophysical probe (103)
Chang’e 7	Lander, rover, mini-flying probe	Mass spectrometers, ground-penetrating radar, Raman spectrometer (104)
PROSPECT instrument package	Lander	Mass spectrometers, permittivity sensor (105)
Canadian Lunar Rover	Rover	Lyman-alpha imager, neutron and gamma ray spectrometer (106)

The quality of in situ analysis strongly depends on the extent to which the sample can be preserved in its original state. Especially for cold-trapped volatiles, such as water, the sample acquisition poses a significant risk with respect to thermal, mechanical, and chemical alteration. Loss processes affect the volatile concentration, distribution, form, and isotope ratio. These effects must be understood and minimized, and unavoidable alterations must be calibrated in advance. Regarding isotopic fractionation, Mortimer et al. (107) showed that for water ice at –75 °C with an initial hydrogen isotope ratio of δD ≈ –76‰ (where δD = [(D/H)_sample_/(D/H)_SMOW_ – 1] × 1,000; SMOW: standard mean ocean water), up to approximately 50% of the original concentration can be lost before the isotope ratio in the remaining sample changes significantly, i.e., more than 10‰. It should be noted that this change heavily depends on the initial D/H ratio and that for lower δD values, such as solar wind hydrogen with δD ≈ −1,000‰, it is much lower when expressed in the delta notation. Furthermore, the fractionation behavior changes significantly in a mixture of water with regolith compared to pure water. Moores et al. (108) showed that a higher amount of dust in the mixture results in a larger isotope fractionation with the preferential release of H_2_O. Therefore, to minimize fractionation and enable the correct interpretation of the origin of hydrogen species, the original composition must be preserved as much as possible. This mainly necessitates adapting the sampling process to avoid any thermal disturbance of the sample caused by interaction with the instrument. However, unknown parameters, such as the thermal conductivity of the potentially ice-bearing soil, its particle size distribution and porosity, or the thermal environment above the surface, pose a significant uncertainty for predicting the thermal behavior during sample acquisition.

Determining these parameters to better estimate the volatile loss is not always possible, as such measurements are often not the primary purpose of the respective instrumentation. While most of the future missions will be equipped with sensors to measure the temperature of the ground-penetrating hardware or the soil (Table 2), these measurements will not necessarily be suited to deduce the thermal properties of the granular regolith. This is very challenging due to the thermal resistance between a respective sensor and the regolith and the thermal-mechanical disturbance induced by the sensor itself. The thermal conductivity of lunar regolith ranges from approximately 10^−4^ to 10^−2^ W m^−1^ K^−1^, as determined by in situ measurements (109) and laboratory analysis of returned samples (e.g., refs. 110 and 111). To derive the thermal properties of the regolith, it needs to be actively or passively heated while monitoring the heat input and temperature over time. This is typically done using the line heat source technique with a thin, needle-like sensor (112). Larger geometries, such as cylindrical probes and drills, can also be used but exhibit a larger disturbance of the soil and potentially more potential for measurement errors due to uneven heat transfer along the probe axis. Grott et al. (113) showed that the thermal conductivity determined by the Apollo lunar heat flow experiment was overestimated by a factor of two to three in the vicinity of the probe due to soil compaction. In addition, it was found that the measurements should have lasted much longer (100 to 200 h) to compensate for this disturbance and sample enough undisturbed regolith to provide a better estimate of the thermal conductivity.

## Conclusion

The science and exploration of the lunar water cycle is one of the main themes of current lunar research, as it has major implications for studying the interactions of airless bodies with the space environment, the alteration of the natural lunar environment due to surface activity, the development of instrumentation for future missions, and the exploration and potential utilization of water as a resource. Understanding the full complexity of the lunar water cycle is only possible through new in situ investigations, analysis of returned samples, analog studies, and simulation of the various source, sink, and loss or conversion processes involved. While individual elements of the lunar water cycle can be reproduced by numerical simulations and experiments, connecting different physical domains and scales is a persistent challenge. A significant problem is the large uncertainty regarding the interactions of hydrogen species and the regolith, including their retention and loss via surface reactions, transformations between H, H_2_, OH, and H_2_O, and the physical and heat and mass transfer properties of ice-bearing regolith. Subsequently, current predictions of the lunar ice stability are based on simplified models, likely underestimating the total abundance of locations favoring cold trapping of lunar water.

More research is needed to reduce the uncertainty of water–regolith interactions, including the determination of temperature- and coverage-dependent sorption kinetics, rates of photodesorption and photodissociation, and sputtering yields. Likewise, the properties of regolith with variable ice content and its implications for heat and mass transfer need to be characterized, including the particle size and morphology, porosity, and tortuosity, and the resulting thermal and gas diffusion coefficients. On a larger scale, the influence of topography and variable shadowing on the migration of water across the surface and within the subsurface needs to be understood to improve current predictions of ice stability and explain localized enhancements in water concentration.

Several lunar surface missions are currently planned, most of them dedicated to the exploration and characterization of water occurrences. One of the main challenges to overcome for these missions is the accessibility of volatile-bearing cold traps due to the extreme thermal environment. For their scientific investigations, the risk of sample disturbance and loss of volatile species during sample acquisition and analysis might limit the quality of the results. To minimize the overall uncertainty in estimating the volatile loss, it is important to monitor sample properties along the process and use complementary sensing and analysis techniques to compensate for different sources of error.

## Data Availability

There are no data underlying this work.
